# Innovation in entrepreneurship during the time of COVID-19: a scoping review of the scientific evidence from Peru

**DOI:** 10.12688/f1000research.134588.1

**Published:** 2023-06-13

**Authors:** Víctor Hugo Fernández-Bedoya, Monica Elisa Meneses-La-Riva, Josefina Amanda Suyo-Vega, Rosario Violeta Grijalva-Salazar, Johanna de Jesús Stephanie Gago-Chávez, Hitler Giovanni Ocupa-Cabrera, Sofía Almendra Alvarado-Suyo, Giovanni Di Deus Ocupa-Meneses

**Affiliations:** 1 Grupo de investigación "Sostenibilidad", Universidad Cesar Vallejo, Los Olivos, Lima, Peru

**Keywords:** Innovation, Entrepreneurship, COVID-19, Peru, Review

## Abstract

**Background:** Entrepreneurship involves the actions of designing, launching and managing a business that initially starts small and grows along with the human structure that integrates it. The health crisis caused by coronavirus disease 2019 (COVID-19) had negative effects on health, but also on business; many ventures had to innovate in order to survive in this uncertain environment. Peru is a country located in Latin America, recognized for its high levels of entrepreneurial self-efficacy, so this scooping review sought to identify the experiences of innovation in entrepreneurship in times of COVID-19 in that country.

**Methods:** We explored the Scopus and Scielo databases for records detailing innovation in entrepreneurship in both English and Spanish. Inclusion and exclusion criteria were: published between March 11, 2020, to May 5, 2023; English, Spanish, and Portuguese language; original articles that present both quantitative and qualitative results; within Peru; articles with an assigned issue and volume number. The search results identified 5 Peruvian experiences that met the research objectives.

**Results:** The records identified deal with innovations in social entrepreneurship, women’s entrepreneurship, entrepreneurship in the educational sector, and new business tools applied during the COVID-19 pandemic. The sources where these records were disclosed were South American journals (3 cases) and conference proceedings (2 cases). The language of the articles was recorded, identifying that most of them are written in Spanish (official language of Peru).

**Conclusions:** We recommend the regional scientific community to disseminate the results of their research in scientific journals indexed in high-level databases in order to have greater visibility.

## Introduction

Entrepreneurship is known as the action involved in the process of designing, launching and managing a business. ^1^^,^^2^ Such a business usually starts as a very small enterprise that makes available to customers the sale of a product, service or process.

On the other hand, the health crisis caused by the coronavirus disease 2019 (COVID-19) pandemic had diverse effects not only on people’s health, but also on the economy. ^3^ Some businesses, due to their nature, had to stop, such as those related to leisure and travel. ^4^ Even businesses related to gastronomy were able to operate with limited seating capacity. ^5^^,^^6^ However, many other businesses had to innovate and look for new alternatives to stay in operation.

According to information from the Pan American Health Organization, on March 11, 2020, the World Health Organization declared a COVID-19 pandemic, due to the high number of cases in 112 countries outside China. ^7^

In Peru, on March 5, 2020, the first imported case of COVID-19 was confirmed in a person with a history of travel to Spain, France and the Czech Republic. From that date until July 31, 2022, samples have been processed for 33,131,204 people with COVID-19, resulting in 3,909,870 confirmed cases, 29,221,334 negative cases and 214,303 deaths. ^7^

During COVID-19 in Peru, many businesses sought to reinvent themselves through innovation, adopting strategies that allowed them to survive the pandemic and even improve their services in the post-COVID-19 scenario and thus innovating. ^8^^–^^11^

Innovation in the business field is known as the strategic process through which a company introduces new products, services or ways of doing things. ^12^ This innovation can be radical (creating something new) or incremental (improving something that already exists). ^13^^–^^15^ Business innovation is essential to a company’s long-term growth and survival as it helps maintain a competitive advantage and meet changing customer needs. ^16^^,^^17^

In this sense, it is necessary to identify the experiences of innovation in entrepreneurship in times of COVID-19 in Peru. This country is located in South America, a region known to have high levels of entrepreneurial effectiveness. ^18^^,^^19^

The objective of this scooping review was to identify the experiences of innovation in entrepreneurship in times of COVID-19 in Peru. In addition, we sought to identify the language in which it was written. Finally, we sought to identify the conclusions of each experience explored.

## Methods

In order to achieve the research objectives, a scooping review (also known as mapping reviews) was conducted, which aims to identify nature and extent of research evidence (usually including ongoing research). ^20^ We followed the Preferred Reporting Items for Systematic reviews and Meta-Analyses extension for Scoping Reviews (PRISMA-ScR) Checklist ^21^ to ensure comprehensive and transparent reporting of our scoping review methodology and findings (see *Reporting guidelines* section below ^27^).

We scanned the Scopus database, and performed the search equation TITLE-ABS-KEY (“entrepreneurship” “COVID-19” “innovation”) AND (LIMIT-TO (AFFILCOUNTRY, “Peru”)). This means that we searched only for articles with the words entrepreneurship, COVID-19, and innovation in the title, abstract or keywords. Based on the articles found, an additional filter was performed, this time by country, identifying only articles registered in the metadata as coming from Peru. This same procedure was used for searches in Spanish, which is one of the official languages of Peru, by means of the search TITLE-ABS-KEY (“ *emprendimiento*” “COVID-19” “ *innovación*”) AND (LIMIT-TO (AFFILCOUNTRY, “Peru”)).

Next, we set out to explore the Scielo database, which is a Brazilian initiative that facilitates the scientific dissemination of research in South America. We searched in English (entrepreneurship AND innovation AND COVID-19) and Spanish ( *emprendimiento* AND *innovación* AND COVID-19), and then selected Peruvian experiences.

After identifying the accessible articles, we proceeded to define the eligibility criteria, taking the following into consideration:

a) Temporality: Due to the recognition of the COVID-19 virus as a pandemic by the WHO from March 11, 2020, to May 5, 2023, we considered these publication dates.
b) Language: We considered papers written in English, Spanish, and Portuguese, as these are the most commonly used languages for scientific dissemination in Latin America, and particularly in Peru.
c) Article type: We considered original articles that present both quantitative and qualitative results on specific innovation experiences in entrepreneurship.
d) Temporal context: Our objective was to identify experiences of innovation in entrepreneurship in times of COVID-19 in Peru, so we only included articles that detail experiences in Peru.
e) Publication status: We only considered completed articles with assigned issue and volume numbers.

The initial results allowed us to identify 11 records extracted from Scopus and 2 from Scielo, totaling 13 in all. This can be seen in Table 1.

**Table 1.** Initial results.

Search code	Database	Language	Initial results
SCO-ENG	Scopus	English	9
SCO-SPA	Scopus	Spanish	2
SCI-ENG	Scielo	English	1
SCI-SPA	Scielo	Spanish	1

Subsequently, we proceeded to download the records to which we had access. In the case of the Scopus database, the documents were downloaded directly from the platform or through the publisher; if possible (we used the institutional access granted by Universidad César Vallejo). On the other hand, for Scielo, which is a database that provides open access articles, all the identified articles were downloaded directly from its platform. Subsequently, the articles were coded, and the extracted data was processed using Microsoft Excel 2021. Finally, 9 records were successfully downloaded.

Then, 2 records were removed due to exclusion criteria: even though the metadata reported it was located in Peru, the content of them didn’t mention any experience from there.

Additionally, because 4 different searches were used, 2 duplicate records were identified, leaving a total of 5 articles included in the scooping review.

**Figure 1.**
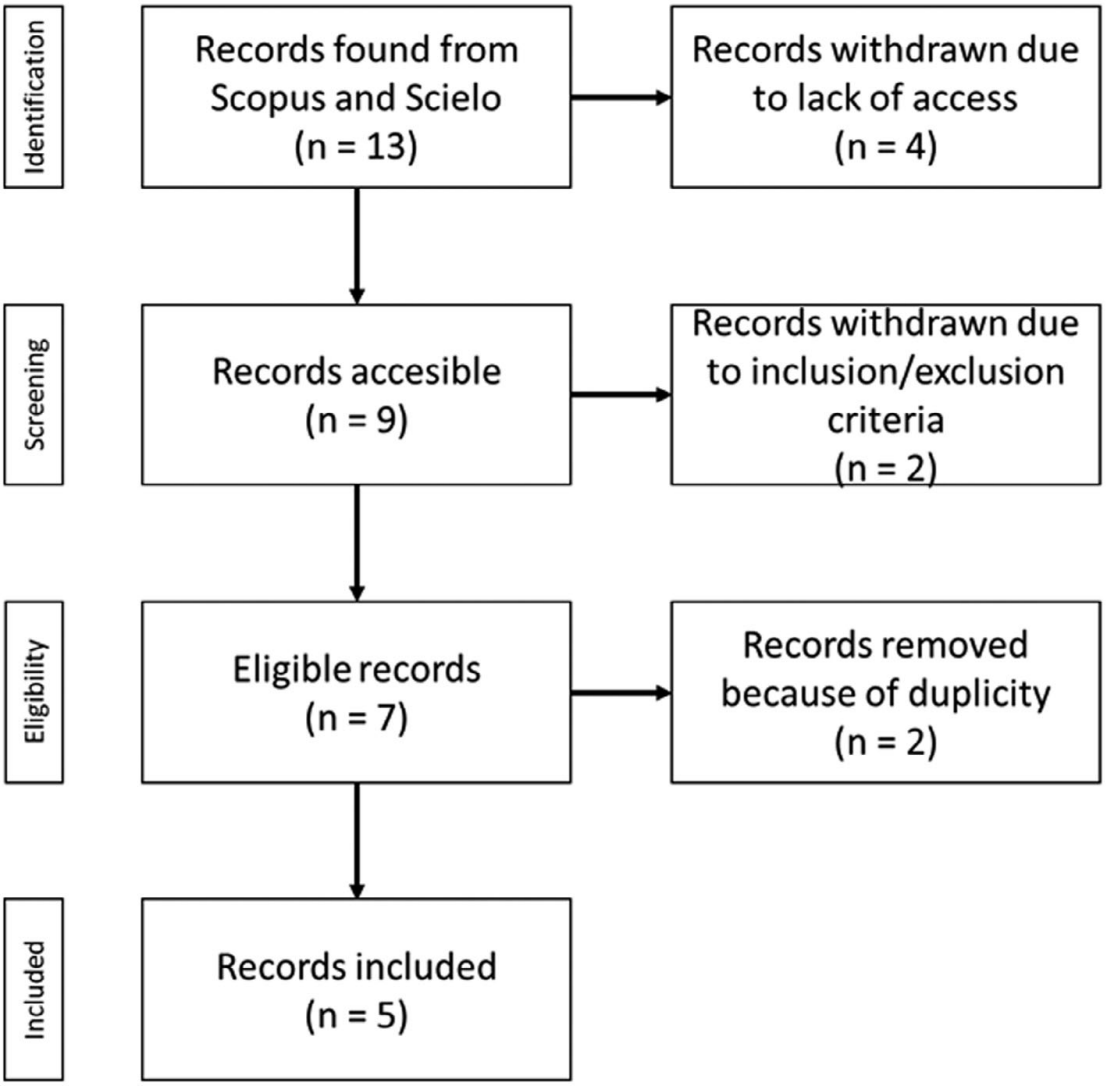
PRISMA flow chart.

Finally, each of the final records identified was reviewed. The process followed can be seen in Figure 1, which shows the PRISMA flow chart.

### Data charting

*Data extraction*: A systematic search was conducted in databases such as Scopus and Scielo, utilizing specific search equations and filters. Relevant articles were identified based on the inclusion criteria aligned with the research objectives.

*Document download*: Following the identification of accessible articles, the records were downloaded. In the case of Scopus, downloads were obtained directly from the platform or through the publisher, utilizing institutional access provided by Universidad César Vallejo. For Scielo, an open-access database, all identified articles were downloaded directly from the platform.

*Data coding and processing*: The downloaded articles underwent coding, and the extracted data was processed using Microsoft Excel 2021. A customized template was developed to ensure systematic extraction and organization of pertinent data from the articles. Please refer to the additional data file for details.

*Data verification and confirmation*: The analysis and findings were based on the information presented within the articles themselves. The focus of the study was on identifying and synthesizing the articles that met the predefined inclusion criteria, rather than conducting primary data collection or additional verification processes. We relied on the data and information provided by the authors in the articles to conduct our analysis and draw conclusions.

## Results

In order to identify the experiences of innovation in entrepreneurship in times of COVID-19 in Peru, we explored the Scopus and Scielo databases.

The 5 records that responded to the research objective were identified. They are “Innovación y emprendimiento social como estrategia para afrontar la Pandemia COVID-19, ^22^ “Peruana del bicentenario: promotora del emprendimiento en tiempos de crisis”, ^23^ “Post COVID-19 Global Macrotrends in the pedagogical practice to achieve Student Outcomes-ICACIT”, ^24^ “Experience In A Training Program To Strengthen Technological Entrepreneurship Through Technological Tools During The Covid-19”, ^25^ and “Generation of New Ventures in the Face of the New Normality: Approaches from its Ethical-Social Dimension”. ^26^ Their code, title and source can be seen in Table 2.

**Table 2.** Experiences of innovation in entrepreneurship in times of COVID-19 in Peru.

Reference number	Title	Source
^22^	Innovación y emprendimiento social como estrategia para afrontar la Pandemia COVID-19	Revista de Ciencias Sociales
^23^	Peruana del bicentenario: promotora del emprendimiento en tiempos de crisis	Comuni@cción: Revista de Investigación en Comunicación y Desarrollo
^24^	Post COVID-19 Global Macrotrends in the pedagogical practice to achieve Student Outcomes-“ICACIT”	2020 IEEE International Symposium on Accreditation of Engineering and Computing Education (ICACIT)
^25^	Experience In A Training Program To Strengthen Technological Entrepreneurship Through Technological Tools During The Covid-19	Proceedings of the LACCEI international Multi-conference for Engineering, Education and Technology
^26^	Generation of New Ventures in the Face of the New Normality: Approaches from its Ethical-Social Dimension	Revista de Filosofia (Venezuela)

In order to identify the experiences of innovation in entrepreneurship in times of COVID-19 in Peru, we explored the Scopus and Scielo databates.

**Figure 2.**
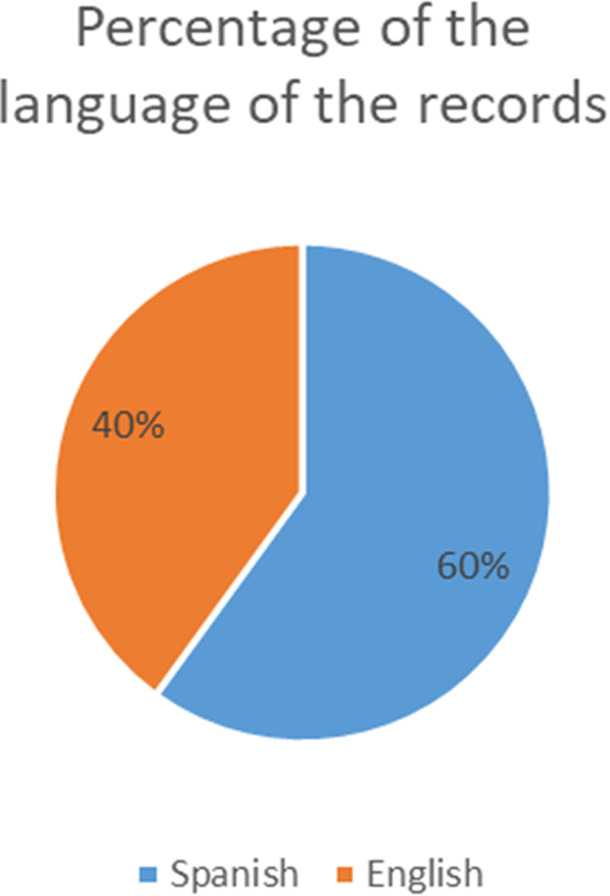
Percentage of the language of the records.

It was also important to identify the language in which each experience was written. Peru is a country whose mainly official language are Spanish, so it was surprising to find that 2 of the 5 records identified were written in English, considered a foreign language. This is shown in Table 3 and Figure 2.

**Table 3.** Language of the records.

Reference number	Language
^22^	Spanish
^23^	Spanish
^24^	English
^25^	English
^26^	Spanish

Finally, the results of each of the experiences identified as records are presented in Table 4.

**Table 4.** Conclusions of the records.

Reference number	Conclusions
^22^	On the one hand, the challenges and social problems in Peru are not few and unfortunately not superficial, COVID-19 is wreaking havoc: it has hit the economy as well as the productive apparatus. In the midst of all this, the need arises to provide answers, as well as solutions to the economic situation suffered by many people who have become unemployed and are looking for a viable alternative economic livelihood. Hence, various forms of social entrepreneurship arise based on social innovation, as a result of business innovation.
^23^	In the case of women entrepreneurs in Peru, it was identified that they have adapted to technology to make their ventures visible and assert their creative skills, knowledge and innovation in products and services. With this, they discovered a diversity of possible activities to add more value to their enterprises from their homes, even in their free time.
^24^	The education sector was hard hit by the health crisis. Due to restrictive measures for the prevention of contagion, they were forced to migrate to virtuality and apply innovation megatrends. The authors mention 10 megratrends: one cross-cutting macrotrend (public awareness), eight macrotrends in the pedagogical practice (disrupting education, assessment of progress, co-creation, UX-focused education, gamification, harness innovation, instantaneous entrepreneurship), and one macrotrend of support (networking and technology).
^25^	The university sector in Peru had to close its physical doors after the social distancing measures decreed by the government. In the case of Universidad Catolica de Santa Maria, the use of digital tools such as social networks, videoconferences, and virtual rooms (Habbo) was implemented, achieving a great reception by students of the various professional careers of the University.
^26^	Entrepreneurship is a collaborative force for social progress, even more so when it is carried out responsibly. In the Peruvian case, ventures are strengthened by innovation through the appropriate use of non-traditional sales channels such as e-commerce, advertising on social networks, online job training, among other aspects. Beyond adapting to the “new normality”, they have overcome the challenges to reinvent themselves, implementing resources, accelerating tests, and launching product design prototypes in record time.

## Discussion

Only 5 records were identified that met the research objectives. It is important to point out that although it is true that the scientific literature reinforces the fact that Latin America is one of the regions with the highest entrepreneurial self-efficacy, there is not much scientific information detailing experiences of innovation in entrepreneurship in Peru.

As for the sources from which the information was extracted, it is worth noting that 60% (3 cases) come from Latin American journals, while the rest (40%, 2 cases) were the result of scientific dissemination in conference proceedings format. This reinforces what was said in the previous paragraph, in relation to the low visibility of Latin American authors specialized in this subject.

The language in which the scientific article was written was also identified. Although it is true that 3 records were written in Spanish and 2 in English, 100% of them had abstract and keywords written in English (even if the remaining content was in Spanish), this shows the will of the authors to obtain more visibility by the international public in search engines. Finally, as for the conclusions of each of the records evaluated, all of them detail limitations due to the “new normality” to which both suppliers of goods/services and consumers had to adapt.

At the national level, the emergence of social entrepreneurship based on social innovation stands out. ^22^ In the case of women entrepreneurs, it is recognized that the social distancing measures and thus running their businesses from home gave them the opportunity to learn new techniques and apply them to add greater value to their ventures. ^23^

A similar case occurred in education. The third register ^24^ details 10 megatrends employed by this type of institutions at the national level; again, innovation in entrepreneurship is present through the implementation of new good practices. The fourth record ^25^ illustrates the case of a Peruvian university that found it necessary to give virtual classes for more than two years, where to break the monotony, they used digital tools such as social networks, videoconferences, and virtual rooms (Habbo).

The latest record ^26^ details how entrepreneurs were forced to take their businesses to the next level through innovation, ventured into new sales channels such as e-commerce, trained their employees virtually for the first time, and ventured into online advertising. All this contributed to their survival and consolidation as a company in that difficult moment of uncertainty.

This study employed a comprehensive search strategy utilizing the Scopus and Scielo databases to identify relevant articles on the topic of innovation in entrepreneurship during the COVID-19 pandemic in Peru. The search equation used specific keywords and filters, ensuring a focused and targeted approach. Additionally, English, Portuguese and Spanish languages were considered, which are widely used for scientific dissemination in Latin America and Peru.

While the selected databases provide a wealth of information, it is important to acknowledge that this research may not have captured every possible relevant article. However, the chosen databases are reputable and widely recognized sources of scholarly literature, increasing the credibility and validity of the findings.

The eligibility criteria were carefully designed to include original articles presenting both quantitative and qualitative results on innovation experiences in entrepreneurship. This rigorous approach ensured that only high-quality, completed articles with assigned issue and volume numbers were considered, minimizing the risk of biased or incomplete data.

The temporal context of the study encompassed the period from March 11, 2020, to May 5, 2023, aligning with the official declaration of COVID-19 as a pandemic by the World Health Organization. This timeframe provided a comprehensive overview of the innovative efforts in entrepreneurship during this challenging period.

While the study focused specifically on Peru, it is important to note that the findings may have implications for other regions facing similar circumstances. The experiences and insights gained from the Peruvian context can serve as valuable references for policymakers, entrepreneurs, and researchers globally.

We recommend that Latin American researchers contribute to the bibliographic collection detailing experiences of innovation in entrepreneurship, especially in the Peruvian case. Very little specific information on these cases was identified in the Scopus and Scielo databases. We make available to the international community the findings of this article. On occasions, the literature (particularly that written in Spanish) is overlooked and not considered; therefore, we have seen fit to present the conclusions of each record identified in the results section. Finally, we encourage a greater number of review articles on this subject, using new search equations, or exploring other local databases that are not very visible at the international level.

## Data Availability

All data underlying the results are available as part of the article and no additional source data are required. Zenodo: PRISMA-ScR Checklist for ‘Innovation in entrepreneurship during the time of COVID-19: a scoping review of the scientific evidence from Peru’. https://doi.org/10.5281/zenodo.7996424. ^27^ Data are available under the terms of the Creative Commons Attribution 4.0 International license (CC-BY 4.0).
