# Supplementary material for: Innovation in entrepreneurship during the time of COVID-19: a scoping review of the scientific evidence from Peru
Source: F1000Res. 2023 Jun 13;12:665. [Version 1] doi: 10.12688/f1000research.134588.1 (PMC11739704; doi:10.12688/f1000research.134588.1)
Supplement: Peer review history

# Reviewer report: Reviewer response for version 1

**DOI:** 10.5256/f1000research.147653.r350225

**Published:** 2025-01-16

**Authors:** Muzaffar Asad

**Affiliations:** 1 Tecnologico de Monterrey, Monterrey, Nuevo Leon, Mexico

Dear Researchers, I appreciate your efforts, you have conducted research on a very interesting topic related to micro small and medium enterprises post COVID because during COVID several firms have faced closure or have faced severe losses which has jeopardized the survival and contribution of the sector. Moreover, you have focused on the green practices which is another major contribution. However, I have realized that while developing the introduction section and justification of the study to show the need for research you have overlooked some important research works which I believe that you must consult to make the paper equipped with more current research in the field. The inclusion of the suggested papers will not only help you to stress over the gaps that calls for further research but also may help you in understanding about the argument that you have built over the topic. Thus, in the introduction and especially, in the Literature where you have written the writeup for the Hypothesis, it is too brief, its better to increase by reviewing more literature and adding detailed discussion over the topic. I strongly recommend you to read and cite and especially criticize the below mentioned studies.

 Asad M, et al., 2024 (Ref 1)

 Kanaan O, et al., 2024 (Ref 2)

 Satar M, et al., 2024 (Ref 3)

 Ta’Amnha M, et al., 2024 (Ref 4)

 Additionally, in the discussions and conclusions section if you link your findings with the latest research and show separately the practical as well as theoretical contribution of the study, it gives a better impression to the reader and improves the readability of the study.

 Finally, a good research is one that opens the horizons for new research. Thus, you are suggested to add a separate subheading for limitations faced and recommendations for the future researchers in the same field.

Are the rationale for, and objectives of, the Systematic Review clearly stated?

Yes

Is the statistical analysis and its interpretation appropriate?

Yes

If this is a Living Systematic Review, is the ‘living’ method appropriate and is the search schedule clearly defined and justified? (‘Living Systematic Review’ or a variation of this term should be included in the title.)

Yes

Are sufficient details of the methods and analysis provided to allow replication by others?

Yes

Are the conclusions drawn adequately supported by the results presented in the review?

Yes

Reviewer Expertise:

Entrepreneurship

I confirm that I have read this submission and believe that I have an appropriate level of expertise to confirm that it is of an acceptable scientific standard, however I have significant reservations, as outlined above.

## References

[1] : Green entrepreneurial leadership, and performance of entrepreneurial firms: does green product innovation mediates?. *Cogent Business & Management* .2024;11(1) : 10.1080/23311975.2024.2355685 10.1080/23311975.2024.2355685

[2] : A mediated moderated analysis of knowledge management and stakeholder relationships between open innovation and performance of entrepreneurial firms. *Uncertain Supply Chain Management* .2024;12(4) : 10.5267/j.uscm.2024.5.028 2383-2398 10.5267/j.uscm.2024.5.028

[3] : The Moderating Role of Entrepreneurial Networking between Entrepreneurial Alertness and the Success of Entrepreneurial Firms. *Sustainability* .2024;16(11) : 10.3390/su16114535 10.3390/su16114535

[4] : Moderating role of technological turbulence between green product innovation, green process innovation and performance of SMEs. *Discover Sustainability* .2024;5(1) : 10.1007/s43621-024-00522-w 10.1007/s43621-024-00522-w

# Reviewer report: Reviewer response for version 1

**DOI:** 10.5256/f1000research.147653.r225120

**Published:** 2024-01-27

**Authors:** Mercy E.I. Ogbari

**Affiliations:** 1 Department of Business Management, Covenant University, Ota, 112233, Nigeria

The study titled Innovation in Entrepreneurship during the Time of COVID-19: A Scoping Review of the scientific evidence from Peru is contemporary and gives the view that locales are opening up to the realities of the present times.

 the study is commendable. the objectives are well stated.

 the study is a short paper however, there were no details of concepts regarding the major constructs.

 the authors did not specifically underpin any theoretical inclination to the study, and neither was emphasis made to prove that such studies have been expanciated upon.

 on the methodology aspect, it's quite clear it's a semantic review, I didn't see much of the evidence on the discussion as it relates to the innovation/ technological entrepreneurship being dealt with in the article.

 the discussions were not detailed, I was wondering what exactly the issues of innovativeness as unveiled at Peru were as regards the highlights of the discussion.

 its summary is not in-depth as the essence of the study is still hidden.

 it would be nice if the authors critically revealed the innovative entrepreneurship engaged at during the COVID-19 in Peru.

 the communication is clear, and the language technicality is ok.

Are the rationale for, and objectives of, the Systematic Review clearly stated?

Yes

Is the statistical analysis and its interpretation appropriate?

Yes

Are sufficient details of the methods and analysis provided to allow replication by others?

Partly

Are the conclusions drawn adequately supported by the results presented in the review?

Partly

Reviewer Expertise:

Entrepreneurship and entrepreneurship education, business strategy, strategic mgt, General Business & Management, Innovation and technology.

I confirm that I have read this submission and believe that I have an appropriate level of expertise to confirm that it is of an acceptable scientific standard, however I have significant reservations, as outlined above.

# Reviewer report: Reviewer response for version 1

**DOI:** 10.5256/f1000research.147653.r178661

**Published:** 2023-06-23

**Authors:** Patricia Haas

**Affiliations:** 1 Universidade Federal de Santa Catarina, Florianópolis, State of Santa Catarina, Brazil

The article entitled "Innovation in entrepreneurship during the time of COVID-19: a scoping review of the scientific evidence from Peru", a systematic review, presents attributes of scientific cooperation and has indications of strengthening the contribution of informative data regarding the pandemic. 

 The review is appropriate for publication and within the expected scope for this type of research. 

 It includes enough information and discussions, constructed in a meaningful way so that the article can be accepted. The Objective is aligned with the title and discussion and the finalization contemplates the content of the study. The references used are sufficient and current.

 The research reinforces and helps clarify the health crisis caused by the coronavirus disease 2019 (COVID-19) with evidence of negative effects on health and business, which reflects the need for innovation to remain in the market. Peru is a country located in Latin America, recognized for its high levels of entrepreneurial self-efficacy, so this Scoping Review sought to identify innovation experiences in entrepreneurship in times of COVID-19 in that country.

 The research reflects the reality of Peru, located in Latin America, but also contributes to the post-pandemic reality of countries of the same size, thus highlighting experiences of innovation in entrepreneurship in times of COVID-19.

 It is noteworthy that the research responds to current questions, is elaborated in the recommended protocols and presents a relevant scientific contribution.

Are the rationale for, and objectives of, the Systematic Review clearly stated?

Yes

Is the statistical analysis and its interpretation appropriate?

Yes

Are sufficient details of the methods and analysis provided to allow replication by others?

Yes

Are the conclusions drawn adequately supported by the results presented in the review?

Yes

Reviewer Expertise:

Public health

I confirm that I have read this submission and believe that I have an appropriate level of expertise to confirm that it is of an acceptable scientific standard.
